# Structural Models for the Dynamic Effects of Loss-of-Function Variants in the Human SIM1 Protein Transcriptional Activation Domain

**DOI:** 10.3390/biom10091314

**Published:** 2020-09-12

**Authors:** Mathew A. Coban, Patrick R. Blackburn, Murray L. Whitelaw, Mieke M. van Haelst, Paldeep S. Atwal, Thomas R. Caulfield

**Affiliations:** 1 Department of Cancer Biology, Mayo Clinic, Jacksonville, FL 32224, USA; Coban.Mathew@mayo.edu; 2 Department of Laboratory Medicine and Pathology, Mayo Clinic, Rochester, MN 55905, USA; blackburn.patrick@mayo.edu; 3 Department of Molecular and Cellular Biology, University of Adelaide, Adelaide SA 5000, Australia; murray.whitelaw@adelaide.edu.au; 4 Department of Clinical Genetics, Amsterdam UMC, University of Amsterdam, Meibergdreef 9, 1105 AZ Amsterdam, The Netherlands; m.vanhaelst@amsterdamumc.nl; 5 Department of Clinical Genetics, Amsterdam UMC, Vrije Universiteit Amsterdam, De Boelelaan 1117, 1081 HV Amsterdam, The Netherlands; 6 Center for Individualized Medicine, Mayo Clinic, Jacksonville, FL 32224, USA; DrA@atwalclinic.com; 7 Atwal Clinic, Jacksonville, FL 32224, USA; 8 Department of Clinical Genomics, Mayo Clinic, Rochester, MN 55905, USA, MN, USA; 9 Department of Neuroscience, Mayo Clinic, Jacksonville, FL 32224, USA

**Keywords:** single-minded homologue 1, enhanced molecular dynamics, computational modeling, variants of uncertain significance, Prader-Willi-like syndrome

## Abstract

Single-minded homologue 1 (SIM1) is a transcription factor with numerous different physiological and developmental functions. SIM1 is a member of the class I basic helix-loop-helix-PER-ARNT-SIM (bHLH–PAS) transcription factor family, that includes several other conserved proteins, including the hypoxia-inducible factors, aryl hydrocarbon receptor, neuronal PAS proteins, and the CLOCK circadian regulator. Recent studies of HIF-a-ARNT and CLOCK-BMAL1 protein complexes have revealed the organization of their bHLH, PASA, and PASB domains and provided insight into how these heterodimeric protein complexes form; however, experimental structures for SIM1 have been lacking. Here, we describe the first full-length atomic structural model for human SIM1 with its binding partner ARNT in a heterodimeric complex and analyze several pathogenic variants utilizing state-of-the-art simulations and algorithms. Using local and global positional deviation metrics, deductions to the structural basis for the individual mutants are addressed in terms of the deleterious structural reorganizations that could alter protein function. We propose new experiments to probe these hypotheses and examine an interesting SIM1 dynamic behavior. The conformational dynamics demonstrates conformational changes on local and global regions that represent a mechanism for dysfunction in variants presented. In addition, we used our ab initio hybrid model for further prediction of variant hotspots that can be engineered to test for counter variant (restoration of wild-type function) or basic research probe.

## 1. Introduction

The single-minded homolog 1 (SIM1) is a founding member of a subset of basic helix-loop-helix (bHLH) proteins that also contain eriod (Per)-aryl hydrocarbon receptor nuclear transporter (ARNT)-SIM (PAS) domains. PAS domain proteins are universally expressed [1], and they have an eclectic array of functions, such as chemotaxis, phototropism, ligand binding, and signal transduction [1]. SIM1 is imperative for development and functionality of paraventricular neurons of the hypothalamus [2], but it is also expressed in kidney and muscle, where its functions have yet to be investigated. As evidence for the importance of SIM1, homozygous null mice are perinatally lethal [3]. Furthermore, SIM1 heterozygous mice develop juvenile obesity and associated diabetes-like symptoms via the etiological mechanism of metabolic imbalance through hyperphagia and lack of compensatory increase in energy output [3,4]. This phenotype is consistent with a crucial role for SIM1 in the leptin-melanocortin signaling pathway that promotes satiety via activation of paraventricular hypothalamic (PVH) neurons. SIM1 can form a heterodimer with either aryl hydrocarbon receptor nuclear translocator (ARNT) or ARNT2 in in vitro cell-based assays but is hypothesized to preferentially dimerize with ARNT2 in vivo. As ARNT2 is enriched in neurons, and *Arnt2*^−/−^ mice phenocopy the loss of PVH neurons observed in *Sim1*^−/−^ mice, it is hypothesized that disruption of the SIM1/ARNT2 axis drives obesity in humans with or without clinical features that are also described in Prader-Willi-like (PWL) syndrome. There are numerous pathogenic missense variants of *SIM1*, though for many, the mechanism by which they exert dysfunction is unknown. A major contributing factor to this is the lack of a full-length, high-resolution experimental structure for SIM1, especially in complex with the aforementioned proteins.

Prader-Willi Syndrome (PWS) is a genetic disorder that is characterized by neonatal hypotonia and feeding problems, short stature, small hands and feet, impaired intellectual development, and from early childhood onwards, hyperphagia, which often results in obesity and diabetes. PWS is caused by lack of expression of genes on the paternally inherited chromosome 15q11.2-q13 region [5]. Pathogenic *SIM1* variants often present with overlapping clinical features (hypotonia, hyperphagia, and variable degree of intellectual deficit), hereafter termed Prader-Willi-like (PWL). However, other *SIM1* variants are not associated with PWL features, but instead are only associated with early onset obesity with variable penetrance. It is currently unclear why certain *SIM1* variants result in PWL symptoms and others do not.

In this study, we sought to identify the mechanism by which several missense *SIM1* variants effectuate pathological conditions through computational modeling, molecular dynamics simulations (MDS), and subsequent analyses. To that end, we generated an original atomistic model of SIM1 dimerized with ARNT, described vide infra, and developed computational/analytical protocols that can be utilized to assess additional pathological variants in future studies. Many structural studies are static, e.g., investigating an X-ray crystal structure. While such information is invaluable, it does have limitations and caveats. The crystal growth and data collection conditions are typically exceedingly artificial, generally with high salt and protein concentrations, extremely viscous, and at cryogenic temperatures. This may result in artificial interactions that can obfuscate the biologically relevant ones. Furthermore, proteins function dynamically, not statically, and a complete understanding of them cannot be achieved in a time-independent examination. With our MDS, we are able to replicate physiological conditions and model how single point mutations can have large effects on the conformations and interactions of proteins, in the context of their innately dynamic natures.

The specific variants we investigated were T46R, D707H, G715V, and D740H. Residue 46 is located in the bHLH domain and interfaces with ARNT. Variant T46R shows severe loss of activity in reporter assays with both the ubiquitous ARNT1 and neuron-enriched ARNT2, and is associated with obesity, though patients lack other PWL symptoms [6]. The remaining three mutants all reside in the SIM C-terminal domain, which, by homology, is hypothesized to be involved with SIM1’s transcriptional regulatory activity [7]. D707H had a moderate impact on transcriptional activity with ARNT1 and ARNT2 and was associated with obesity [8]. Similarly, G715V had a moderate impact on activity with ARNT1 and ARNT2 and was associated with obesity in at least two unrelated patients [9]. D740H actually had a modest increase in activity with ARNT1 but not ARNT2; patients presented with obesity, but no other PWL symptoms [6]. We chose these variants in order to discern the tripartite relationship between transcriptional activation (functional), the associated SIM1 variants (structural), and time-dependent behavior of the proteins (dynamical).

## 2. Materials and Methods

### 2.1. Computer-Assisted Modeling of SIM1 and ARNT Protein Structures

The sequence of human SIM1, a cellular protein encoded by the single-minded family bHLH transcription factor 1 gene (*SIM1*), was taken from the National Center for Biotechnology Information (NCBI) Reference Sequence NM_005068.2. There is no complete SIM1 experimental structure. There are, however, a number of partial structural models, as well as domains with good sequence and presumably structural similarity with crystallized homologs. Amino acid (aa) residues 336–766 were used to generate a model of the single-minded 1 C-terminal domain. The SIM1 sequence was constructed based on many alignments, in which each domain was modeled as separate units, which were subsequently assembled in composite for a final hybrid model. Segments were taken from Protein DataBank identification PDB IDs codes: 1WA9, 3H82, 4F3I, 4H6J, 4WN5, 4ZP4, 4ZPH, 4ZPR, 4ZQD, and 5SY5, and additional ab initio modeling was used for filling in loop regions with programs such as Yasara (loop-builder scanning PDBs) and iTasser [10,11,12,13,14,15,16,17]. The human ARNT sequence was taken from the NCBI Reference Sequence NM_111111.1. Likewise, there is no complete ARNT experimental structure, but there are partial structures, as well as homologs like HIF-2a:ARNT and CLOCK:BMAL1, which have different segments taken from PDB IDs 1X0O, 2A24, 2K7S, 4EQ1, 4LPZ, 4EQ1, 5V0L, 5Y7Y, and 5SY7. Likewise, additional ab initio modeling was used for filling in loop regions.

The modeling was built as a hybrid model from consensus between the programs PRIME (Prime v3.0, Schrodinger, LLC, New York, NY, USA) [18,19], YASARA SSP/Homology/PSSM Method [10,11,12,13,14], and TASSER [15,16,17]. The variable loops and gaps were filled using knowledge-based homology and knowledge-based potentials with YASARA, or the ab initio approach of TASSER, Porter/Distill, RaptorX, and ORCHESTRAR [15]. Each missing loop was modeled using the Loop Search module implemented in Sybyl 8.0 or with the YASARA loop modeler [10,11,12,13,14,20,21,22]. Only loops with the highest homology and lowest root-mean-square-deviations (RMSDs) were selected for the final models. The side chains and rotamers were adjusted with knowledge-based potentials, simulated annealing with explicit solvent, and small equilibration simulations using YASARA’s refinement protocol and verified by WHAT-IF and PROCHECK [23].

Dividing the protein into overlapping groups, based on hundreds of generated separate protein fragments, we constructed 27 structural templates from structural homology, secondary structure prediction, or ab initio modeling methods. These fragments were allowed to generate in overlapping regions and with or without added loops for undefined regions. Combined fragments were overlaid using in-house superposition algorithms to determine optimal overlay and energies [24,25,26,27,28,29], which left the extraneous overlaid residues to be discarded as unnecessary. For example, both YASARA and PRIME models were used to make a hybrid model of superior Z-scoring from all the generated all-atom models. These templates comprise a composite model that is derived from the various methods used to generate the fragments, thus each model was kept for possible conformations to consider the C-term region. Algorithms, like TASSER, were considered on fragments with low homology and the entire length protein for comparison. Full-length TASSER models never generated Z-scores (default value); however, individual fragments gave reasonable structures, which were retained for conformational comparison.

Refinement of the fragments was completed using either YASARA refinement module or NAnoscale Molecular Dynamics (NAMD2) protocols. These refinements started with the Secondary Structure Prediction (SSP) for YASARA parameterization, pKa assignment, solvation and simulated annealing, and pre-equilibration setup (minimizations) [10,11,12,21]. Both homology and fold recognition were considered and a final refinement with the entire model was completed using YASARA for 250 ps of molecular dynamics (MD) using knowledge-based force fields. Additionally, YASARA supports an extensive and large loop library for modeling loops and gaps [21]. The superposition and subsequent refinement of the overlapping regions yields a complete model with two interesting separated larger global regions (Domains 1–5) for SIM1. The final C-terminal domain was subjected to energy optimization with Polak-Ribiere conjugate gradient (PRCG) with an R-dependent dielectric.

All model conformations were verified with WHAT-IF and PROCHECK and have valid conformations consistent with good phi-psi space [13]. In addition, we verified important features from the generated structures, examining the packing (two- and three-dimensional (2D and 3D)), dihedrals, PhiPsi, Backbone, and Packing3 (WHAT-IF implementation), plus forcefield considerations. Atom consistency was checked for all 766 amino acids, verifying correctness of chain name, dihedrals, angles, torsions, non-bonds, electrostatics, atom type, and parameters. Each model was exported to the following formats: Maestro (MAE) and YASARA (PDB). Model manipulation was done with Maestro [24], or Visual Molecular Dynamics (VMD) [25].

After model construction and validation were completed, SIM1 was protein–protein docked to ARNT using Glide. Docking was carried out using the homolog structure generated as described above, and scoring was assessed compared to this template, using the standard PIPER protocols with 30,000 generated samples and >30 retained complexes ranked by fit [24], which allows complete translational-rotational and side chain adjustment mode via default settings. The top 30 accepted poses were inspected, subjected to energy minimization with OPLS3 forcefield, and further filtered for those with lowest energy in Molecular Mechanics/Generalized Born Surface Area (MM/GBSA) evaluation.

### 2.2. Molecular Dynamics Simulations

Molecular dynamics (MD) was completed on each model for conformational sampling. The primary purpose of MD, in this scenario, is examining any conformational variability that may occur with different dimerization pairs, such as ARNT2. Briefly, each SIM1 system was minimized with relaxed restraints, using either steepest descent (SD) or PRCG, and equilibrated in solvent with physiological salt conditions. More detailed descriptions of our particular MD methodology are discussed in the literature [26,27,28,29]. The protocol for refinement included the following steps: (1) minimization with explicit water molecules and ions, (2) energy minimization of the entire system, and (3) MDS for >10 ns to relax to the forcefield (OPLS3/Amber) [30,31]. Following the refinement protocol, production simulations were completed to collect data, with an additional MD production length of >500 ns. This overall production length included the contribution of various MD runs for adequate sampling of the dynamical phenomena (during MD refinement/setup), and each long (>100 ns) production runs. As these production runs revealed significant structural changes resultant from the point mutations, they were sufficient to accomplish our study goal. We reasonably suspect that even longer simulations would reveal even greater structural rearrangements; however, we already identified sufficient mechanistic details that explicate the detrimental nature of these SIM1 variants with our MDS sampling methods.

OPLS3 (Desmond)/Amber (NAMD2) forcefields were used with the current release of the NAnoscale Molecular Dynamics 2 engine [31,32]. The simulated system, including hydrogens, consisted of ~2.0 × 10^5^ atoms with solvation using SPC/E water and ions. In all cases, we neutralized with counter-ions, and then created a solvent with 150 mM Na^+^ Cl^−^ to recreate physiological strength. SPC/E water molecules were added around the protein at a depth of 15–18 Å from the edge of the molecule, depending upon the side [33]. Our protocol has been previously described in the literature [29]. Simulations were carried out using the particle mesh Ewald technique with repeating boundary conditions with a 9 Å nonbonded cut-off, using SHAKE with a 2 fs timestep.

Pre-equilibration was started with three stages of minimization with 10,000 steps of SD, PRCG, and relaxing restraints, then followed by 1000 ps of heating under MD, with the atomic positions of nucleic and protein fixed. Then, two cycles of minimization (5000 steps each) and heating (1000 ps) were carried out with soft restraints of 10 and 5 kcal/(mol·Å^2^) applied to all backbone atoms and metals. Next, 5000 steps of minimization were performed with solute restraints reduced to 1 kcal/(mol·Å^2^). Following that, 400 ps of MDS were completed using relaxing restraints (1 kcal/(mol·Å^2^)) until all atoms are unrestrained, while the system was slowly heated from 1 to 310 K using velocity rescaling upon reaching the desired 310K during this equilibration phase. Additionally, Isothermal–isobaric ensemble (NPT) equilibration was established using velocity rescaling for >10 ns. Finally, production runs of MD were carried out with constant pressure boundary conditions (relaxation time of 1.0 ps) for over 500 ns. A constant temperature of 310 K was maintained using the Berendsen weak-coupling algorithm with a time constant of 1.0 ps. SHAKE constraints were applied to all hydrogens to eliminate X-H vibrations, which yielded a longer simulation time step (2 fs). Our methods for equilibration and production run protocols are in the literature [27,34,35,36]. Translational and rotational center-of-mass motions were initially removed. Periodically, simulations were interrupted to have the center-of-mass removed again by a subtraction of velocities to account for the “flying ice-cube” effect [37]. Following the simulation, the individual frames were superposed back to the origin, to remove rotation and translation effects.

## 3. Results

**Figure 1:**
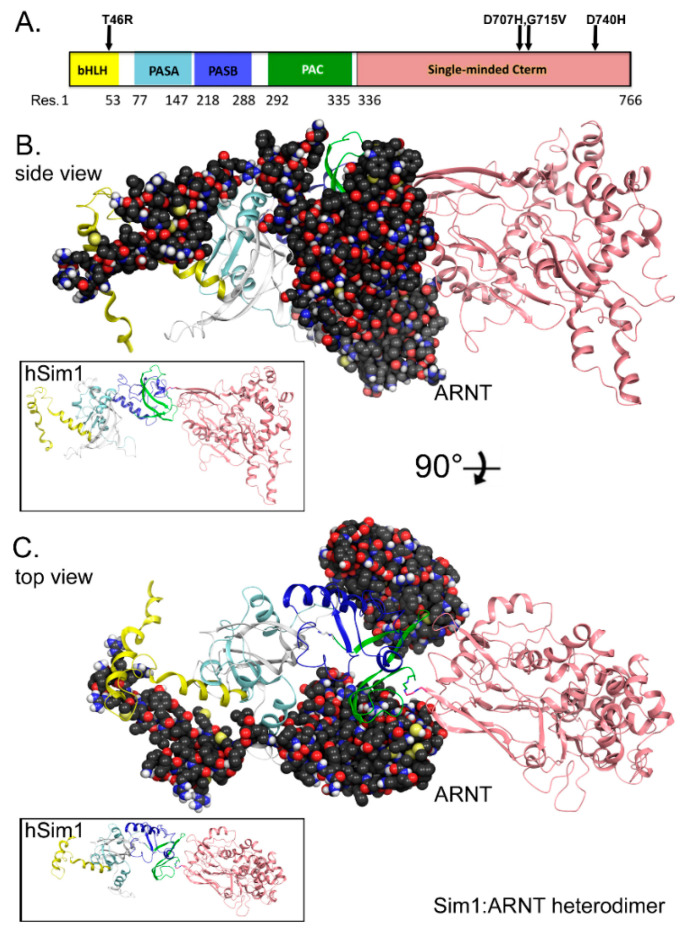
Generation of a composite hybrid (with mixed ab initio) model for full-length SIM1. (**A**) Domain legend for human SIM1. The domains are colored and key variants labeled above. (**B**) Dimeric model for the SIM1: ARNT structures. Full-length (766 aa) SIM1 is shown in ribbons colored as shown in the domain legend. ARNT structure is shown in van der Waals (VdW) spheres with carbon in dark-gray, oxygen-red, nitrogen-blue, and polar hydrogens-white. Inset is shown without ARNT in same orientation for reference. (**C**) Same dimeric SIM1: ARNT structure rotated 90° along the X-axis.

A complete full-length, 766 amino acid structural model for human SIM1 protein was generated. The protein consists of the bHLH, PASA, PASB, PAC, and single-minded C-terminal domains, which are covered by residues 1–53, 77–147, 218–288, 292–335, and 336–766, respectively (Figure 1A). The gap regions formed from residues 54–76, 148–217, and 289–291 are loop-rich and have low homology to catalogs of existing structures. Our structural methods have been previously described [27,38,39,40]. We implemented our modeling technologies [27,38,39,40] to achieve the first all-atom structural model for SIM1 dimerized with the ARNT crystal structure (Figure 1B,C). The ARNT structure was reported at 2.63 Å resolution for residues 98–142, 159–228, 259–271, 301–315, 334–346, and 360–464, with gaps in the reported PDB for residues 143–158, 229–258, 272–300, 316–333, and 347–359, respectively [41]. Our ARNT structure is based solely on the available X-ray structures and used to generate contact data for the ARNT: SIM1 heterodimer (Figure 1B,C). Molecular dynamics simulations were completed on several variants of SIM1 with known pathogenicity (T46R, D707H, G715V, and D740H) (Figure 1A). Inset images on Figure 1B,C reveal the top and side view for SIM1 in its apo form.

### 3.1. SIM1 and ARNT Heterodimer Interface Has Contacts Consistent with Stabilizing Interface

We observed numerous strong contacts between ARNT and the SIM1 heterodimer structure. Additionally, we found many soft contacts that contribute favorably to the interface. For strong contacts, the H-bond 2.2–2.5 Å and 20° angle is considered strong, while 2.6–3.2 Å is considered a soft contact. The default is set at 2.5 Å for our cutoff, which is technically a strong contact. Section 3.1.1. through Section 3.1.5. discuss a domain-by-domain analysis of the dimer contacts.

#### 3.1.1. SIM1 bHLH Domain

**Figure 2:**
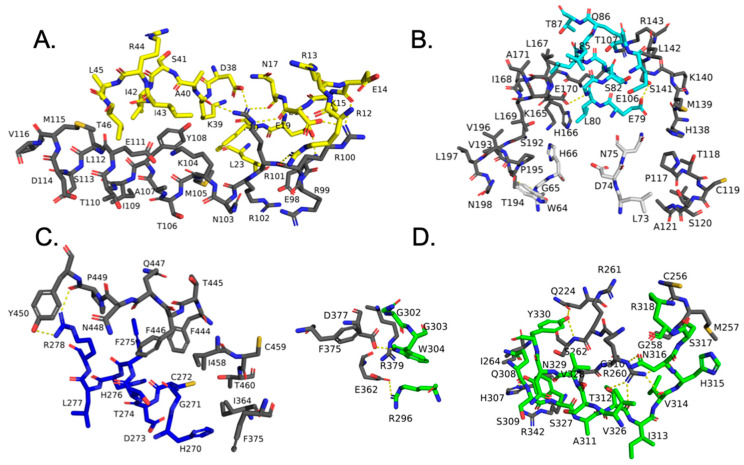
Modeling of the ARNT: SIM1 heterodimeric complex interaction interfaces. SIM1 domain carbons are colored per Figure 1A and residues labeled. Polar contacts between SIM1 and ARNT are shown as yellow dashes. Global context of the complex is given in Figure 1. (**A**) bHLH SIM1 domain interactions with ARNT are displayed, as described in Section 3.1.1. The SIM1 bHLH domain is colored yellow and ARNT is given in black carbons. (**B**) PASA SIM1 domain interactions with ARNT are presented, as described in Section 3.1.2. The SIM1 PASA domain is colored cyan or white and ARNT is given in black carbons. (**C**) PASB SIM1 domain interactions with ARNT are shown, as described in Section 3.1.3. The SIM1 PASB domain is colored blue and ARNT is given in black carbons. (**D**) PAC SIM1 domain interactions with ARNT are exhibited, as described in Section 3.1.4. The SIM1 PAC domain is colored green and ARNT is given in black carbons.

For the SIM1 bHLH domain, ARNT forms contacts for strong interaction pairs between ARNT: R100 side chain nitrogen (NH1) to SIM1:Glu16 side chain oxygen (O_ε2_), ARNT: E98 backbone nitrogen (N_H1_) to SIM1:E19 side chain oxygen (O_ε2_), and ARNT: R101 side chain nitrogen (N_H1_) to SIM1:D38 side chain (O_δ2_). While, the soft contacts for the SIM1 bHLH domain of the ARNT:SIM1 heterodimer include ARNT residues E98, R99, R100, R101, R102, N103, K104, M105, T106, Y108, E111, L112, D114, M115, and V116, and SIM1 residues R12, E14, E16, N17, S18, E19, F20, L23, A24, L27, D38, K39, A40, I42, I43, L45, T46, and L50, respectively (Figure 1B,C and Figure 2A).

#### 3.1.2. SIM1 PASA Domain

The SIM1 PASA domain forms strong interactions with ARNT via ARNT: E163 side chain oxygen (O_ε1_) to SIM1:H83 side chain nitrogen (N_δ2_), and ARNT: S141 side chain oxygen (O_g_) to SIM1:E79 side chain oxygen (O_ε2_). Soft contacts for the SIM1 PASA domain of the ARNT:SIM1 heterodimer include ARNT residues P117, T118, A121, L122, H138, M139, K140, S141, L142, R143, L159, T160, Q162, E163, H166, L167, A171, A172, D191, S192, V193, T194, P195, V196, and N198, and SIM1 residues W64, G65, H66, L73, N75, R78, E79, L80, G81, S82, H83, L84, Q86, T87, L88, E106, T107, and Q116, respectively (Figure 1B,C and Figure 2B).

#### 3.1.3. SIM1 PASB Domain

For the SIM1 PASB domain, strong contacts occur between ARNT: R278 side chain (N_H1_) to SIM1:P449 backbone carbonyl oxygen (O). ARNT forms soft contacts with the SIM1 PASB domain through ARNT residues I364, S365, R366, F375, V376, D377, H378, Q387, I458, F446, Q447, N448, P449, and Y450, and SIM1 residues H267, H270, G271, C272, T274, F275, and R278, respectively (Figure 1B,C and Figure 2C).

#### 3.1.4. SIM1 PAC Domain

ARNT forms strong interaction pairs with the SIM1 PAC domain between ARNT: E362 side chain (O_ε2_) to SIM1:P449 backbone carbonyl oxygen (O), and ARNT: D377 side chain (O_d2_) to SIM1:W304 side chain (N_ε1_). While, the ARNT:SIM1 PAC domain soft contacts include ARNT residues S259, R260, R261, S262, F263, I264, H307, C308, T309, G310, and Y311, and SIM1 residues R296, G302, G303, W304, Q308, Y310, A311, T312, V314, H315, N316, S317, R318, V326, S327, Y330, and T335, respectively (Figure 1B,C and Figure 2C,D).

#### 3.1.5. SM Domain of SIM1 and ARNT

**Figure 3:**
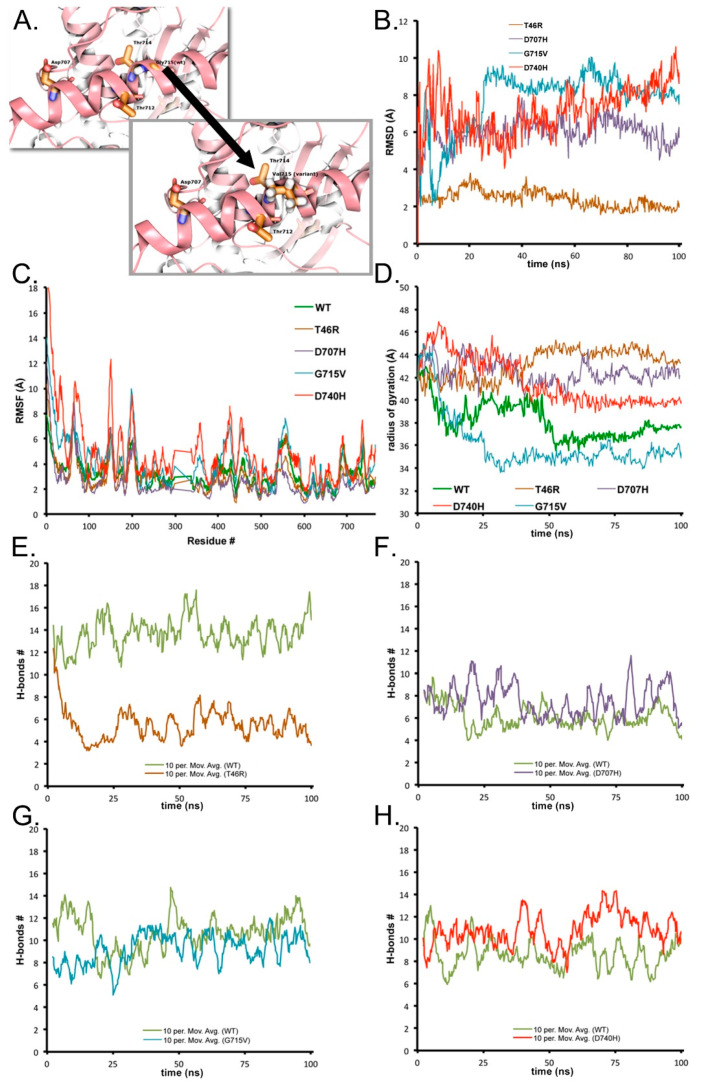
Analytical metrics for decomposition of SIM1 variants versus WT behavior. (**A**) Key example for a pathogenic variant, G715V (panel 1 and 2 showing before and after variant). Colored by domain and rendered in ribbons and licorice sticks for adjacent amino acids. (**B**) Local root-mean-square-deviation for each variant is given, where the local region is defined as all residues within 8 Å of the selected variant. (**C**) Per residue root-mean-square-fluctuation over entire simulation timeframe (>500 ns combined). (**D**) Radius of gyration measurement for each protein as a measure of the particles’ size over time. (**E**) T46R: Amount of hydrogen bonding present within an 8 Å cutoff of T46R versus the same amount of H-bonds present in WT during simulation. (**F**) D707H: Amount of hydrogen bonding present within an 8 Å cutoff of D707H versus the same amount of H-bonds present in WT during simulation. (**G**) G715V: Amount of hydrogen bonding present within an 8 Å cutoff of G715V versus the same amount of H-bonds present in WT during simulation. (**H**) D740H: Amount of hydrogen bonding present within an 8 Å cutoff of D740H versus the same amount of H-bonds present in WT during simulation.

The SM domain of SIM1 and ARNT possess strong interaction pairs among ARNT:E223 side chain oxygens O_ε1_ and O_ε2_ to SIM1:T481 side chain (O_g1_) and SIM1:Q753 side chain (N_ε2_) respectively, ARNT:S259 side chain oxygen (O_g_) (via hydrogen) to SIM1:E336 side chain (O_ε1_), and ARNT:D219 side chain (O_δ2_) to SIM1:Y447 side chain hydroxyl (O_H_). While, the soft contacts for SIM1 SM domain of the heterodimer include ARNT residues D219, G258, K220, R222, Q224, S226, M255, C256, M257, and E312, and SIM1 residues E336, G480, T481, Y477, A752, Q753, H755, K756, and G757, respectively (Figure 1B,C and Figure 2D). These contacts are given in greater mechanistic detail in the following section on analysis measurements (Figure 3) and in the Supplemental Materials.

### 3.2. SIM1 Dynamics Demonstrates Pathogenic Variants Have Both Local and Global Effects

**Figure 4:**
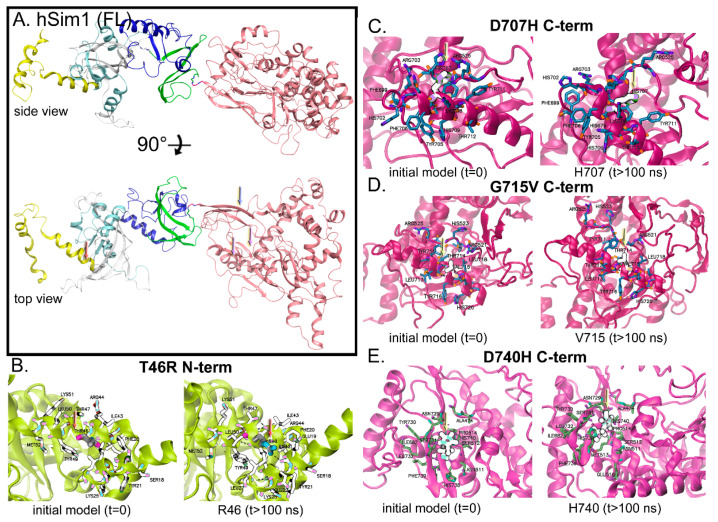
Molecular dynamics simulations demonstrate reorganization of the pathogenic site on SIM1. (**A**) The monomeric, unbound form of SIM1 shown with the same two orientations from Figure 1. Rendered in ribbons and colored by domain. (**B**) T46R mutant at zero time and after 100 ns. (**C**) D707H mutant at zero time and after 100 ns. (**D**) G715V mutant at zero time and after 100 ns. (**E**) D740H mutant at zero time and after 100 ns. Ribbons colored by domain and residues within 8 Å of the mutant are shown in licorice stick rendering. The mutant residue is shown in exaggerated Goodsell rendering.

**Figure 5:**
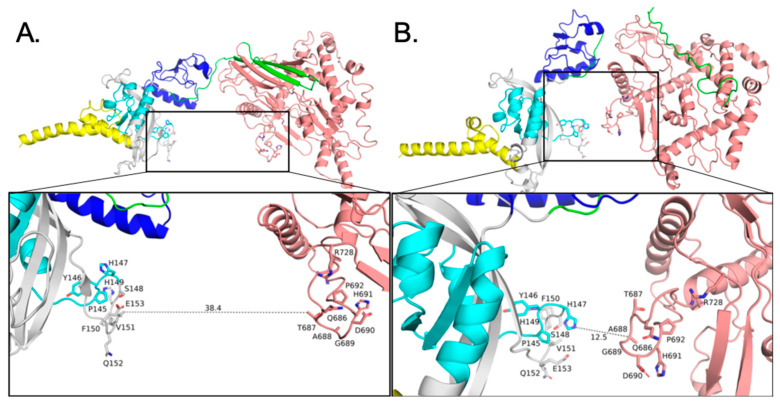
Closure of space between PASA domain and SM domain in WT SIM1 during apo simulation. The structure is colored by domain (as per Figure 1A) and rendered in ribbons and licorice sticks for residues of interest. (**A**) SIM1 in its apo, unbound form is shown for the initial starting conformation. Call-out box shows a close-up of the PASA domain and SM domain, with a 38.4 Å gap where ARNT would occupy. (**B**) SIM1 in its apo, unbound form is shown after completion of >100 ns simulation. Call-out box shows a close-up of the same region, now approximately 26 Å closer. This closed conformation would preclude ARNT from binding.

The two most illustrative poses for SIM1 are shown in Figure 4A (namely, side and top view). The placement of the pathogenic variants is either on the far N-terminal side (position 46) or well into the C-terminal end of the SM domain (positions 707, 715, 740) (Figure 4A–E). A simulation for the full-length wild-type (WT) SIM1 (apo) was completed (Supplementary Movie S1). Simulations were performed on apo SIM1 to assess the impact that the variant-mutation would induce on the conformational dynamics prior to association with ARNT. Variations in the conformational presentation would thereby reduce the likelihood of proper SIM1: ARNT interface. We believe a mechanistic investigation of the variants would be biased by the presence of ARNT pre-dimerized with SIM1 in the simulations, failing to reveal important structural reorganizations influencing the interface. The central region (residues 300–335) is not displayed here, in order to reveal interesting possible interactions between the N-terminal regions and C-terminal regions of SIM1 in its apo form (Figure 5 and Supplementary Movie S1). Global measurements are given and discussed in Section 3.2.1. through Section 3.2.4.

#### 3.2.1. T46R Variant

T46R variant engenders some reorganization, likely due to replacing the polar uncharged threonine to the positively charged arginine, which has a bulky guanidinium moiety (Figure 4B). Adjacent residues K51, L50, Y49, M52, Y21 K25, I43, R44, and T47 are all affected over the 150 ns simulation by the T46R mutation (Figure 4B and Supplementary Movies S2 and S3). The Supplementary Movies show a side-by-side comparison of the first 300 aa from the WT and R46 SIM1 protein and a zoom into the region within 12 Å of T46. Compared to the other variants, it has the least amount of local alteration.

The next set of variants covers the single-minded C-terminal domain and was simulated for >150 ns (Supplementary Movie S4). Supplementary Movie S4 shows WT versus all four variants in the SM domain, T46R, D707H, G715V, and D740H.

#### 3.2.2. D707H Variant

The D707H variant induces reorganization via substituting negatively charged Asp to positively charged His (Figure 4C). Nearby residues R525, F699, H702, Y705, F706, H707, K708, H709, Y711, and T712 are all disturbed over the 150 ns simulation by the change from the D to H variant (Figure 4C and Supplementary Movie S5). The Supplementary Movie shows a zoom into the 12 Å region surrounding H707 during the simulation.

#### 3.2.3. G715V Variant

The G715V variant propagates reorganization due to the insertion of a hydrophobic moiety (valine) where there was no side chain (Figure 4D). Adjacent residues R521, H523, R525, T711, L713, T714, V715, Y716, and H720 are all influenced over the 150 ns simulation by the change from the G to V variant (Figure 3A and Figure 4D, and Supplementary Movie S5). The Supplementary Movie exhibits a close-up of the 12 Å region surrounding V715 during the simulation. This valine could be particularly upsetting to the helix arrangement (Figure 3A).

#### 3.2.4. D740H Variant

The D740H variant effectuates reorganization likely due to exchanging negatively charged Asp to positively charged His (Figure 4E). Proximate residues A474, N511, S512, P514, I682, N729, Y730, L732, H738, and F739 are all impacted over the 150 ns simulation by the change from the D to H variant (Figure 4E and Supplementary Movie S5). The Supplementary Movie displays the 12 Å region surrounding H740 during the simulation.

### 3.3. Detailed Analyses for Local Deviations in Geometry Lead to Larger Amplitude Changes via Correlated Motions

**Figure 6:**
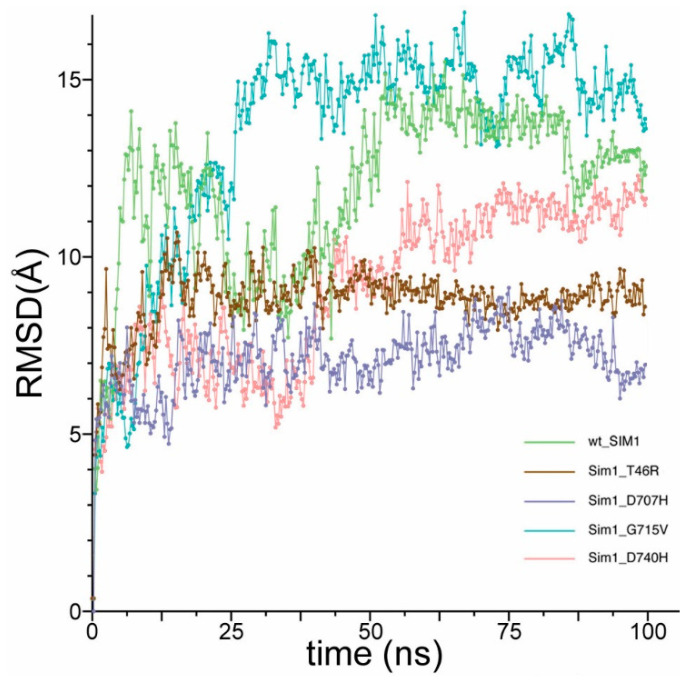
Global root-mean-square-deviation (RMSD), a metric for large-scale conformational changes of SIM1 variants versus WT. Global root-mean-square-deviation for each variant is given, where every residue from the protein is averaged into an accumulated measure of the total amount of change of the structure as a function of time. Colors are given in the figure legend.

The global measure of the change in the entire full-length SIM1 (apo) during molecular dynamics simulations is given (Figure 6). Here, we report that only G715V has a more grossly changed state over the course of the 150 ns simulation. However, we observed interesting apo SIM1 WT motion between N-term and C-term (Figure 5A,B, and Supplementary Movie S1), which could be an unbound stable form of SIM1. Mutant G715V gives RMSD around 15 Å from the starting conformation, having gone into completely different global orientation between the N- and C-term due to the flexibility from the loosened helix. Mutants T46R and D707H showed less RMSD shift than WT (~5 Å difference), while mutant D740H converges with WT after 100 ns of simulation (Figure 4). The global RMSD does show that after approximately 100 ns, each of the simulations settled to a general global conformation, as the RMSD only retains small fluctuations around an average. The initial structure for all of these models was essentially the same, aside from in silico point mutations, and each variant settled to a different average RMSD with respect to this initial state: G715V~15 Å, WT~12 Å, D740H~11 Å, T46R~9 Å, and D707H~9 Å. Because the global comparisons do not address individual residues or other reasons for variants’ loss-of-activity, we pursued multiple other metrics for analysis.

First, local RMSD within 8 Å of the mutant gives a good indication of how much local geometric rearrangement occurs as a consequence of the individual variant, which were measured with respect to the initial frame from the WT structure for SIM1. Mutant T46R has the smallest RMSD change from the set (~3 Å from initial), while H707, V715, and H740 all have large jumps to >6 Å from their initial frames (Figure 3B). Mutant D740 has the biggest change early in the simulation and then the residues settle around 8 Å from initial, while V715 shows the greatest number of large fluctuations ranging from 2–10 Å for the first 45 ns, which corresponds to the helix loosening and destabilization of the SM domain. The H707 mutant has much the same effect as the H740, but lesser amplitude (~6 Å average).

To further assess the effect of the variants on the rest of the structure, a root-mean-square-fluctuation (RMSF) per residue calculation was completed to determine which residues fluctuated the most over the entire time course of the simulation, i.e., time-averaged fluctuation (Figure 3C). While flattened values indicate a region of lower mobility, the larger fluctuating residues indicate a more dynamic structure undergoing rapid changes that can contribute to large conformational changes. Ignoring the trailing tail ends (1–20 and 750–766) is generally prudent when considering RMSF, since the termini unsurprisingly have mobility in excess of other regions of the protein.

Mutant H740 had the largest amplitude changes (6–13 Å), which were in residues 65–75, 150, 169, 199, 338–362, 423–431, 451–452, 556, 689, and 735 (Figure 3C). D740 is located at a partially buried sheet that is tightly neighboring nearby residues. D740 appears optimized in that position, with strong polar contacts to R471, S731, and N729. A histidine in that position makes severe clashes with those and/or other residues, and is also likely repelled by R471. To harbor a histidine, the extensive reordering of nearby regions is evidenced via the various substantial RMSF peaks in proximity to the aforementioned polar contacts. Close behind in amplitude (4–11 Å) was mutant V715, which occurred in residues 43–66, 105–107, 114, 155, 198, 257, 343–368, 406–435, 480, 535–581, 637–638, and 735–742. G715 is located at the partially buried side of a helix, and a valine in that position makes severe clashes with the side chains of Y711 and E719, as well as the backbone and C-beta of H523. The significant structural rearrangements that need to occur to accommodate a valine in that semi-buried portion of helix corroborate the largest global RMSD and significant localized RMSF peaks of that variant. Mutant H707 has only a few peaks (>6 Å) that exceed the WT graph, which occurs at positions 57–76, 144–147, 208, and 545. D707 resides on a helix with nearby residues Q704, K708, R525, and H527, notably mainly basic residues. Interesting, R46 mostly mirrors WT RMSF, but does have a few peaks with different values: position 63–65 (8.84 Å) surpasses WT (~6 Å) and 532–603 is lower than WT by ~2 Å for that entire sequence, and similarly, stays flattened from 682–740. T46 is on the solvent-exposed side of a helix, with fewer residues within reach of the sidechain, and exchanging from a polar to a charged residue in a solvent-exposed and unconstrained area explains the minimal dynamical impact, shown via minimal RMSD and RMSF changes. However, T46 is one of the bHLH residues that makes direct contact with ARNT, therefore an exchange to a bulkier residue arginine is likely the etiology of the pathogenicity of this mutant. In general, regions of elevated RMSF in the variants often correspond to portions of domains that make direct contact with ARNT, as discussed in Section 3.1. The majority of these elevated peaks are not in proximity to the mutations, and therefore are impacted differentially from the motions of the WT SIM1 through an allosteric mechanism, ergo most of the deleterious effects of these variants would not be uncovered via static structural predictions, perhaps except T46R.

### 3.4. Particle Size Changes as a Consequence of the Variant Chosen

Another typical analysis is to examine the global structure’s spatial arrangement or state of compactness. Using a radius of gyration (RoG) calculation (akin to a hydrodynamic radius), we estimate the average distance from the centroid (particle center of mass) to the edges for all atoms in the structure. The RoG can grow or shrink depending on a variety of factors.

Based on earlier observations of the interaction between the N- and C-term from apo SIM1 (Supplementary Movie S1 and Figure 5), it is not surprising to expect that the RoG would collapse over the course of the simulation when plotted versus time. This is precisely what is seen with RoG for the WT sequence (green line) (Figure 3D), which seems to start around 41 Å and stabilize at ~38 Å. Variants R46, H707, and H740 maintain a larger RoG than WT but H740 does collapse to around 40 Å after 55 ns of simulation. However, both R46 and H707 maintain larger RoG at 42 and 44 Å, respectively. Intriguingly, V715 collapses in to around 34 Å after just 25 ns of simulation, thus forming the most compact of the structures. Implications for these compact versus extended conformations may alter the ease of binding to partner proteins such as ARNT. The R46 mutant shows least local deformation but large global reorganization, which may be a function against its activity. 

### 3.5. Stabilization of the Local Region Shifts through Hydrogen Bonding Network Disruptions (Triggering the Correlated Motion Cascade)

Based on the understanding of the local and global changes in structure, examining the shift in the local hydrogen-bonding (H-bond) network versus the WT sequence can be informative for establishing a triggering mechanism that released the conformational change. All H-bonds were measured within an 8 Å cutoff of the residue (and included the entire residue within that cutoff).

Looking at the H-bonds from WT versus R46 reveals an important difference, namely the loss of over 50% of the stabilizing H-bonds (dropped from 14 to 6 H-bonds) (Figure 3E). This loss of H-bonds could explain how the loosened N-term would maintain a larger RoG but still have smaller peaks on RMSF, since it is more unwound but not as interactive as in the C-term variants (Supplementary Movies S2 and S4). Mutant H707 has slightly increased total average number of hydrogen bonds between 8 and 10, whereas WT in the same region is only 4–7. The V715 mutant has a similar trend to H707 with an average just over 10 and WT at 8. Mutant H740 has over 11 H-bonds on average and WT in the same region during the simulation maintains approximately 8. From this list, we can observe that R46 lost 50% while the other variants gained 20–30% hydrogen bonds during the same time interval.

### 3.6. Apo SIM1 Has Room to Move Forming Intra-Molecular Interactions

The effect of the dampened H-bonds in R46 carried over to the intra-domain interactions (N-term to C-term) (Figure 5A,B), where WT has domain interaction and R46 stays extended (not shown). SIM1 residues with a possibility of interaction over the course of a very long simulation include (N-term) H119, P145, Y146, H147, S148, V151, and E153, with (C-term) Q686, T687, D690, H691, P692, and R728. Chemical crosslinking could be conducted on these as a means of abrogating ARNT binding for validation. WT has intrinsic motion to move in this way (Supplementary Movie S1), which may help facilitate binding to ARNT or other important molecules (DNA, etc.).

**Figure 7:**
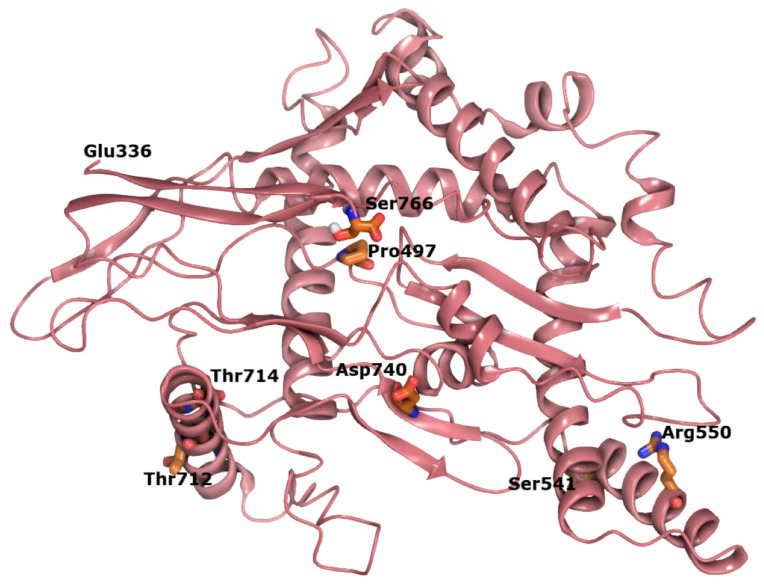
Mapping of all relevant positions in the SM domain (C-term) and their relative position. The SM domain is colored pink in ribbons with key residues in thick licorice rendering for emphasis (carbons-orange, oxygen-red, nitrogen-blue).

In order to examine the spatial relationship between variants in the C-terminal region of SIM1, we constructed an ab initio model of this region, which has not been structurally characterized to date (Figure 7). The p.G715 residue is in a helix that is facing toward solvent from the protein in the single-minded 1 C-terminal domain, which has a plethora of residues as possible interactions (Supplementary Tables S1–S3). Substitution of glycine for valine at this position leads to local increases in hydrophobicity and is predicted to disrupt helix stability over time. Validation of our hybrid model for the SIM1 through generation of a high-quality crystal structure may be useful in mapping additional variant hotspots and in generating hypotheses regarding the functional consequences of pathogenic variants that fall within the transcription regulatory domain.

## 4. Discussion

Several disease-associated variants have been identified in the C-terminal transcription regulatory region around the p.G715V variant identified in this patient, which for this study includes H707D and H740D. We also examined an N-term mutant p.T46R, which has known pathogenicity, but also offers a C-term control group. Lastly, we also studied the WT SIM1 for comparison to all variants.

### 4.1. Mutant T46R

The mutant T46R has pronounced difference in global structure despite minimal differences on the local conformational switching (RMSD/RMSF), but the disrupted H-bond network and highly increased RoG give good indication that the N-term is somewhat de-activated from this variant, since the protein is the most extended of all SIM1 variants. The aforementioned difference is enough that the N- to C-term dynamics are highly dampened and likely, there would be lessened DNA binding capacity or ARNT binding (Figure 3B–E and Figure 4B, Supplementary Movies S3 and S4). In addition, T46 makes direct contact with ARNT, likely a contributing factor as to why this variant had severe impact on ARNT binding.

### 4.2. Mutant H707D

The mutant H707D has a pronounced switch in the local conformational region (RMSD/RMSF), but the disrupted H-bond network is somewhat increased and the RoG is much increased from WT, which may cause the protein to have too much labile motion to properly bind ARNT, cofactors, or DNA. When comparing RMSF for the variants with the WT sequence, the mutant D707 is second most increased, coming after D740. This variant has the second largest RoG (compared with WT) that may contribute to it being too extended to make decent contacts with partner proteins or ARNT binding (Figure 1C, Figure 3B–D,G, and Figure 4D, and Supplementary Movies S3 and S5).

### 4.3. Mutant H740D

The mutant H740D has largest switch in the local conformational region (RMSD/RMSF), with some added H-bond network, and the RoG is only marginally increased from WT, but has huge spikes in RMSF for individual residues from SIM1 that might alter how ARNT binds. The motion for the N- to C-term dynamics is similar to WT but the individual residues with fluctuation give SIM1 unique conformations (Figure 4D) that likely affect how ARNT binds (Figure 1C and Figure 3B–D,H, and Supplementary Movies S3 and S7).

### 4.4. Mutant G715V

Our structural models indicate that G715V would have a detrimental effect on the protein’s function via an altered local structure that perturbs multiple regions of the structure and may perturb the ARNT binding affinity for SIM1. Mutant G715V has minimal changes in the H-bonding of the local region, but large shifts in the local RMSD that come from other interactions discussed above. These new interactions establish a very stable structure that has the lowest RoG from any model in this study. This rather compact state for G715V SIM1 would likely frustrate the ARNT binding and other partner complexes (Figure 3B,D and Figure 4D, and Supplementary Movies S4 and S6). While the C-terminal region of SIM1 does not mediate interactions with ARNT, ARNT2, or DNA, a variant of this region could potentially disrupt or alter recruitment of regulatory co-factors and hence affect function of the SIM1-ARNT2 heterodimer in target gene regulation. However, the identities of these co-factors remain to be determined. Future studies will examine the spatial relationship of this variant and other neighboring variants in the context of a SIM1/ARNT2 heterodimer, which may reveal novel pathological mechanisms of disease.

## 5. Conclusions

In this study, we constructed a full-length model of the SIM1: ARNT dimer. In addition, we performed MDS of apo SIM1 to examine the impact of known pathogenic variants. Of the variants discussed, only T46R makes direct contact with ARNT. Possibly, static structural predictions might explain T46R as pathogenic via alteration of a direct contact, though the disruption of the hydrogen-bonding network and N- to C-term dynamics would be impossible to discern. However, the remaining variants would not be predicted as pathogenic from a simple structural inspection. We propose that the pathogenicity of these variants derives from increasing the conformational flexibility of several regions that make direct contact with ARNT, through a dynamic and allosteric mechanism. The increase in mobility of these binding regions impedes structural pre-configuration, thus explaining the somewhat lesser impact of these variants on ARNT binding, relative to T46R.

## Supplementary Materials

The following are available online at https://zenodo.org/record/4034310#.X2NACYsRVPY and https://www.mdpi.com/2218-273X/10/9/1314/s1, Movie S1: Molecular dynamics simulation for the wild-type human SIM1 full-length, Movie S2: Molecular dynamics simulation for the wild-type human SIM1 and T46R variant for the first 300 amino acids (N-term comparison), Movie S3: Molecular dynamics simulation for the T46R variant of human SIM1, which is only showing residues 1–300 for N-term closeup, Movie S4: Molecular dynamics simulation for the wild-type and four variants for SIM1 (T46R, D707H, G715V, and D740H) for the C-term domain only (SM domain), Movie S5: Molecular dynamics simulation for the D707H variant of SIM1, showing the C-term domain only (SM domain), Movie S6: Molecular dynamics simulation for the G715V variant of SIM1, showing the C-term domain only (SM domain), Movie S7: Molecular dynamics simulation for the D740H variant of SIM1, showing the C-term domain only (SM domain), Table S1: Hydrogen Bonding in Variant T46R, Table S2: Hydrogen Bonding in Variant D707H, Table S3: Hydrogen Bonding in Variant G715V, Table S4: Hydrogen Bonding in Variant D740H. PDBs are available within supplementary material online as well.
