# Supplementary material for: Structural Models for the Dynamic Effects of Loss-of-Function Variants in the Human SIM1 Protein Transcriptional Activation Domain
Source: Biomolecules. 2020 Sep 12;10(9):1314. doi: 10.3390/biom10091314 (PMC7563489; doi:10.3390/biom10091314)
Supplement: Supplementary file 1 [file biomolecules-10-01314-s001.pdf]

Supplementary Information

Structural models for the dynamic effects of loss-of-function variants in the human SIM1 protein transcriptional activation domain

Matt Coban[1], Patrick R. Blackburn[2], Murray L. Whitelaw[3], Mieke M. van Haelst[4,5], Paldeep S. Atwal[6,7], Thomas R. Caulfield[8*]
[1]Department of Cancer Biology, Mayo Clinic, Jacksonville, FL
[2]Department of Laboratory Medicine and Pathology, Mayo Clinic, Rochester, MN
[3]Department of Molecular and Cellular Biology, University of Adelaide, Adelaide, South Australia, Australia
[4]Department of Clinical Genetics, Amsterdam UMC, University of Amsterdam, Amsterdam, The Netherlands
[5]Department of Clinical Genetics, Amsterdam UMC, Vrije Universiteit Amsterdam, Amsterdam, The Netherlands
[6]Center for Individualized Medicine, Mayo Clinic, Jacksonville, FL
[7]Department of Clinical Genomics, Mayo Clinic, Jacksonville, FL
[8]Department of Neuroscience, Mayo Clinic, Jacksonville, FL
*Correspondence: Thomas R. Caulfield, PhD, Department of Neuroscience, Mayo Clinic, 4500 San Pablo Road, Jacksonville, Florida 32224. Phone: +1 904 953 6072; Fax: +1 904 953 6072; E-mail: caulfield.thomas@mayo.edu

S.1. Movie Legend

Movie S1. Molecular dynamics simulation for the wild type human SIM1 full-length. Colored by domain per Figure 1 and showing licorice sticks for relevant residues. Colors are standard (C-gray, N-blue, O-red, H-white, S-yellow) except where highlighted-emphasis carbons are used.

Movie S2. Molecular dynamics simulation for the wild type human SIM1 and T46R variant for the first 300 amino acids (N-term comparison). Rendering style same as in Movie S1.

Movie S3. Molecular dynamics simulation for the T46R variant of human SIM1, which is only showing residues 1-300 for N-term closeup. Rendering style same as in Movie S1.

Movie S4. Molecular dynamics simulation for the wild type and four variants for SIM1 (T46R, D707H, G715V, and D740H) for the C-term domain only (SM domain). Rendering style same as in Movie S1.

Movie S5. Molecular dynamics simulation for the D707H variant of SIM1, showing the C-term domain only (SM domain). Rendering style same as in Movie S1.

Movie S6. Molecular dynamics simulation for the G715V variant of SIM1, showing the C-term domain only (SM domain). Rendering style same as in Movie S1.

Movie S7. Molecular dynamics simulation for the D740H variant of SIM1, showing the C-term domain only (SM domain). Rendering style same as in Movie S1.

S.2. Detailed analysis for each H-bond network region within the local 8 angtrom cutoff for changes due to variant

S2.1. Parameters used in the calculation of hydrogen bonds:

- Atomselection 1: (all) and (name "N.*" "O.*" "S.*" FA F1 F2 F3)

- Atomselection 2: (same residue as all within 8 of resid SELECTION) and (name "N.*" "O.*" "S.*" FA F1 F2 F3)

- Update selections every frame: yes

- Donor-Acceptor distance: 3.0

- Angle cutoff: 20

- Type: pair

Table S1: Hydrogen Bonding in Variant T46R

donor	acceptor	occupancy	donor	acceptor	occupancy
GLY121-Main	ILE101-Main	85.32%	THR47-Side	ILE43-Main	2.26%
THR120-Side	GLN116-Main	60.00%	MET52-Main	SER48-Main	1.61%
SER48-Side	ARG44-Main	49.35%	ARG44-Side	GLY121-Main	1.61%
THR47-Main	ILE43-Main	45.81%	LYS39-Side	ASN17-Side	1.45%
THR120-Main	GLN116-Main	43.06%	ARG44-Side	ASN122-Side	1.29%
MET102-Main	VAL94-Main	42.10%	LYS39-Side	ARG13-Main	1.29%
ALA40-Main	ASP38-Side	39.19%	TYR21-Main	ASN17-Main	0.97%
LYS25-Side	GLU22-Side	25.32%	ILE42-Main	ASP38-Main	0.97%
LYS39-Side	GLU16-Side	24.84%	SER105-Side	TYR103-Side	0.81%
PHE20-Main	GLU16-Main	24.03%	GLN116-Main	SER109-Side	0.81%
ARG46-Main	ILE42-Main	17.42%	GLU16-Main	ARG12-Main	0.65%
ARG44-Main	ALA40-Main	13.87%	ARG44-Side	VAL117-Main	0.65%
PHE56-Main	MET52-Main	10.32%	SER41-Main	ASP38-Side	0.65%
GLU22-Main	SER18-Main	8.23%	SER71-Side	SER67-Main	0.65%
TYR21-Side	SER31-Main	7.74%	GLN116-Side	THR120-Side	0.65%
ILE43-Main	LYS39-Main	7.10%	VAL54-Main	LYS51-Main	0.48%
ALA24-Main	PHE20-Main	7.10%	LYS15-Side	GLU19-Side	0.32%
LEU45-Main	SER41-Main	6.45%	LEU23-Main	PHE20-Main	0.32%
SER41-Side	ASP38-Side	6.45%	TYR21-Side	SER35-Side	0.32%
TYR49-Main	LEU45-Main	6.13%	VAL94-Main	TYR103-Main	0.32%
LYS25-Main	TYR21-Main	5.97%	THR34-Side	PRO30-Main	0.32%
GLU19-Main	LYS15-Main	5.65%	SER41-Side	ASP38-Main	0.32%
LEU23-Main	GLU19-Main	5.65%	ARG44-Side	THR120-Main	0.32%
THR34-Main	PRO30-Main	4.68%	MET52-Main	TYR49-Main	0.32%

Table S2: Hydrogen Bonding in Variant D707H					
**donor**	**acceptor**	**occupancy**	**donor**	**acceptor**	**occupancy**
THR714-Side	ALA710-Main	75.81%	TYR705-Main	HIS702-Main	6.77%
LYS708-Main	GLN704-Main	65.16%	ARG703-Side	GLY700-Main	6.61%
THR714-Main	ALA710-Main	53.23%	LEU718-Main	THR714-Main	5.81%
TYR711-Main	HIS707-Main	48.71%	ARG525-Main	GLU617-Side	4.84%
THR712-Main	LYS708-Main	44.52%	LYS675-Side	ASN697-Main	4.68%
ALA710-Main	PHE706-Main	42.90%	GLY715-Main	TYR711-Main	4.03%
HIS709-Main	TYR705-Main	41.94%	GLU719-Main	GLY715-Main	3.23%
LEU713-Main	HIS709-Main	41.29%	LYS675-Side	CYS698-Main	3.23%
GLY524-Main	GLU617-Side	32.58%	ARG703-Side	ASP529-Side	1.61%
LEU678-Main	ALA674-Main	32.58%	HIS679-Side	CYS698-Main	1.29%
LYS675-Main	LEU671-Main	26.77%	GLY526-Main	HIS707-Side	1.29%
PHE706-Main	HIS702-Main	23.55%	HIS523-Side	THR615-Main	1.13%
LYS675-Side	GLU530-Side	22.10%	HIS709-Side	TYR705-Side	0.97%
HIS520-Side	SER518-Main	21.45%	ARG703-Side	HIS527-Main	0.97%
TYR716-Main	THR712-Main	18.55%	SER680-Side	LYS675-Main	0.81%
LYS675-Side	ASN697-Side	17.74%	ARG521-Side	TYR711-Side	0.65%
ARG521-Side	GLU617-Side	17.42%	ARG521-Side	ILE522-Main	0.32%
ALA717-Main	LEU713-Main	15.81%	LEU671-Main	SER667-Main	0.32%
THR712-Side	LYS708-Main	15.16%	HIS527-Side	ARG703-Main	0.32%

Table S3: Hydrogen Bonding in Variant G715V					
**donor**	**acceptor**	**occupancy**	**donor**	**acceptor**	**occupancy**
ARG296-Main	ASP273-Side	94.85%	THR712-Side	LYS708-Main	3.00%
THR714-Side	ALA710-Main	87.55%	HIS527-Side	ASP707-Side	2.15%
TYR722-Side	GLN483-Main	85.19%	ASP707-Main	ARG703-Main	2.15%
HIS679-Side	CYS698-Main	44.85%	TYR677-Main	LEU673-Main	1.93%
VAL519-Main	GLY491-Main	42.92%	PHE706-Main	HIS702-Main	1.72%
HIS720-Side	GLU725-Side	37.34%	THR714-Main	ALA710-Main	1.72%
LEU718-Main	THR714-Main	35.62%	LEU678-Main	LYS675-Main	1.50%
ALA710-Main	PHE706-Main	34.98%	ARG525-Main	ASP707-Side	1.50%
ARG521-Side	GLU487-Side	34.76%	SER695-Side	HIS679-Main	1.29%
SER492-Side	VAL519-Main	31.76%	ASP723-Main	GLU719-Main	1.29%
ARG296-Side	GLU719-Side	30.04%	SER724-Side	THR481-Side	1.29%
LYS708-Main	GLN704-Main	28.76%	HIS523-Side	THR615-Side	1.07%
GLY526-Main	ASP707-Side	28.11%	VAL618-Main	ALA623-Main	0.86%
LEU298-Main	HIS268-Main	23.39%	HIS520-Side	SER518-Side	0.86%
LEU713-Main	HIS709-Main	22.75%	SER148-Side	ASP723-Side	0.86%
VAL715-Main	TYR711-Main	22.32%	THR615-Side	HIS523-Side	0.64%
GLU725-Main	HIS720-Main	21.24%	SER695-Main	ASP681-Main	0.64%
HIS620-Side	GLY485-Main	21.03%	ASP273-Main	HIS270-Main	0.64%
THR712-Main	LYS708-Main	19.31%	ALA674-Main	SER670-Main	0.43%
ALA717-Main	LEU713-Main	18.45%	HIS709-Side	TYR705-Side	0.43%
HIS520-Side	SER518-Main	11.59%	GLY616-Main	ALA625-Main	0.43%
HIS709-Main	TYR705-Main	10.94%	ALA625-Main	GLY616-Main	0.43%
TYR716-Main	THR712-Main	9.87%	THR481-Side	SER724-Side	0.43%
TYR722-Main	LEU718-Main	9.87%	ASP681-Main	LEU678-Main	0.43%
HIS720-Main	TYR716-Main	9.87%	HIS523-Side	GLU617-Side	0.43%
THR481-Side	LEU721-Main	9.87%	HIS620-Side	GLU487-Side	0.43%
TYR716-Side	PHE150-Main	8.15%	HIS149-Side	HIS276-Side	0.43%
HIS270-Side	GLU719-Side	7.30%	TYR722-Side	PRO482-Main	0.21%
TYR711-Main	ASP707-Main	6.87%	THR693-Main	HIS685-Main	0.21%
TYR255-Side	LEU298-Main	6.65%	ARG728-Side	PRO684-Main	0.21%
LEU721-Main	ALA717-Main	6.01%	SER724-Side	LEU721-Main	0.21%
HIS720-Side	TYR716-Main	5.58%	SER743-Side	ILE727-Main	0.21%
GLU719-Main	VAL715-Main	4.72%	THR274-Main	HIS270-Main	0.21%
HIS523-Side	THR615-Main	4.51%	SER724-Main	LEU721-Main	0.21%
HIS520-Side	ARG521-Main	3.86%	SER724-Side	TYR722-Main	0.21%
TRP490-Side	GLU487-Side	3.43%	LEU678-Main	ALA674-Main	0.21%
HIS270-Main	ARG296-Main	3.43%	HIS149-Side	CYS272-Main	0.21%
HIS685-Side	GLU153-Side	3.43%	ILE516-Main	TYR677-Side	0.21%

Table S4: Hydrogen Bonding in Variant D740H

donor	acceptor	occupancy	donor	acceptor	occupancy
THR714-Side	ALA710-Main	86.70%	LYS708-Main	GLN704-Main	4.08%
THR714-Main	ALA710-Main	67.38%	THR726-Main	GLU725-Side	3.86%
HIS709-Main	TYR705-Main	66.31%	TYR711-Main	ASP707-Main	3.86%
LEU721-Main	ALA717-Main	66.09%	THR712-Main	LYS708-Main	3.00%
GLU725-Main	HIS720-Main	60.52%	GLY526-Main	ASP707-Side	2.58%
LEU678-Main	ALA674-Main	57.30%	HIS720-Main	TYR716-Main	2.58%
TYR722-Side	GLU487-Side	53.43%	ARG521-Side	THR615-Main	2.15%
PHE706-Main	HIS702-Main	48.93%	ARG525-Side	HIS523-Main	2.15%
TYR722-Main	LEU718-Main	48.50%	HIS527-Side	ASP707-Side	1.93%
HIS720-Side	TYR716-Main	43.78%	HIS620-Side	GLU487-Side	1.93%
ALA717-Main	LEU713-Main	40.77%	HIS520-Side	SER492-Side	1.93%
LEU713-Main	HIS709-Main	40.34%	THR712-Side	LYS708-Main	1.72%
TYR716-Main	THR712-Main	38.20%	SER695-Main	ASP681-Main	1.72%
VAL519-Main	ARG746-Main	37.34%	ASP707-Main	ARG703-Main	1.50%
ARG746-Main	ALA517-Main	31.97%	ILE727-Main	GLU725-Main	1.50%
HIS620-Side	GLU719-Side	30.90%	ARG525-Side	ASP707-Side	1.29%
LEU718-Main	THR714-Main	24.89%	TYR711-Side	HIS523-Side	1.29%
ALA710-Main	PHE706-Main	22.96%	HIS709-Side	TYR705-Side	1.29%
HIS515-Main	THR742-Main	16.95%	ARG521-Side	THR615-Side	1.29%
HIS520-Side	ARG521-Main	15.02%	VAL519-Main	SER492-Side	1.07%
GLU719-Main	GLY715-Main	14.16%	THR481-Side	TYR722-Main	1.07%
HIS520-Side	SER518-Main	12.88%	HIS744-Main	HIS515-Main	0.86%
HIS685-Side	GLU725-Side	12.88%	SER492-Main	VAL519-Main	0.64%
ASP723-Main	GLU719-Main	12.45%	ALA674-Main	SER670-Main	0.43%
SER518-Side	ARG493-Main	12.45%	TYR677-Main	LEU673-Main	0.21%
SER492-Side	VAL519-Main	12.23%	ARG525-Side	ASP664-Side	0.21%
ALA517-Main	HIS744-Main	11.37%	THR726-Side	GLU725-Side	0.21%
ARG525-Main	HIS523-Side	7.73%	SER724-Main	LEU721-Main	0.21%
HIS679-Main	LYS675-Main	5.58%	ARG728-Side	PRO684-Main	0.21%
GLY715-Main	TYR711-Main	4.29%	SER695-Side	ASP681-Main	0.21%
